# IL-33 Provides Neuroprotection through Suppressing Apoptotic, Autophagic and NF-κB-Mediated Inflammatory Pathways in a Rat Model of Recurrent Neonatal Seizure

**DOI:** 10.3389/fnmol.2017.00423

**Published:** 2017-12-19

**Authors:** Yuan Gao, Cheng-liang Luo, Li-li Li, Guang-hua Ye, Cheng Gao, Hao-chen Wang, Wen-wen Huang, Tao Wang, Zu-feng Wang, Hong Ni, Xi-ping Chen, Lu-yang Tao

**Affiliations:** ^1^Department of Forensic Medicine, Wenzhou Medical University, Wenzhou, China; ^2^Department of Forensic Medicine, Medical School of Soochow University, Suzhou, China; ^3^Department of Neurology Laboratory, Children’s Hospital of Soochow University, Suzhou, China

**Keywords:** apoptosis, autophagy, IL-33, recurrent neonatal seizure, ST2L

## Abstract

Interleukin-33 (IL-33) is a novel identified chromatin-associated cytokine of IL-1 family cytokines. It signals through a heterodimer comprised of ST2L and IL-1RAcp, and plays a crucial role in many diseases. However, very little is known about the role and underlying intricate mechanisms of IL-33 in recurrent neonatal seizure (RNS). To determine whether IL-33 plays an important regulatory role, we established a neonatal seizure model in this study. Rats were subjected to recurrent seizures induced by inhaling volatile flurothyl. Recombinant IL-33 or PBS were also administered by intraperitoneally (IP) before surgery, respectively. Here, our current results indicated that RNS contributed to a significant reduction in IL-33 and its specific receptor (ST2L) expressions in cortex. While, in hippocampus, RNS induced an increase in IL-33 and ST2L evidently, compared with Sham group. After injection with IL-33, however, a remarkable increase in total IL-33 was detected both in brain cortex and hippocampus. In addition, IL-33 was mainly co-localized in the nuclear of GFAP^+^ astrocytes and the cytoplasm of the Iba-1^+^ microglia and IL-33^+^/NeuN^+^ merged cells. In parallel, ST2L was expressed mainly in the membrane of GFAP^+^ astrocytes, Iba-1^+^ microglia and NeuN^+^ neurons, respectively. Furthermore, administration of IL-33 improved RNS-induced behavioral deficits, promoted bodyweight gain, and ameliorated spatial learning and memory ability. Moreover, IL-33 pretreatment blocked the activation of NF-κB, resisted inflammatory cytokines IL-1β and TNF-α increase, as well as suppressed apoptosis and autophagy activation after RNS. Collectively, IL-33 provides potential neuroprotection through suppressing apoptosis, autophagy and at least in part by NF-κB-mediated inflammatory pathways after RNS.

## Introduction

Epilepsy is the third most common chronic neurologic disease, affecting many people of different ages, especially in childhood. Despite advances in pharmacological and surgical treatments of epilepsy, comparatively little is known about underlying mechanisms and effective therapies of the generation of individual seizures (Berg et al., 1999; Vezzani et al., 2011). Therefore, a greater understanding of the pathologic development has the potential to search novel treatments for epilepsy.

The pathological basis of epilepsy consists of neuronal loss, axonal sprouting and inflammatory responses (Xiao et al., 2015). Especially, inflammation plays a primary role in the pathophysiology of seizures and epileptogenesis (Sayyah et al., 2003; Vezzani and Granata, 2005). After epileptic seizure, a large number of pro-inflammatory cytokines such as IL-1β and TNF-α is produced from astrocytes and microglia activation (Allan and Rothwell, 2001; Vezzani and Granata, 2005). In turn, IL-1β and TNF-α contributed to neuronal hyperactivity, neuronal loss, blood-brain-barrier rupture and ultimately resulted in the likelihood of generation and procession of epilepsy (Vezzani and Granata, 2005; Banks and Erickson, 2010). Thus, epileptic seizures and inflammatory cytokines could form a positive feedback loop between epileptogenesis and inflammation.

As a novel identified chromatin-associated cytokine, IL-33 drives distinct cytokines production in mast cells (MC), invariant natural killer T (NKT) and T helper (Th)_2_ cells (Schmitz et al., 2005; Drube et al., 2010). IL-33 can also induce signaling pathways by binding the heterodimeric receptor complex, which is comprised of ST2 and IL-1R accessory protein (IL-1RAP) and then recruits myeloid differentiation primary-response protein 88 (myD88), IL-1R-associated kinase 1 (IRAK1) and IRAK, eventually leads to the activation of numerous signaling proteins such as NF-κB and MAPK (De la Fuente et al., 2015; Molofsky et al., 2015). In central nervous system (CNS), numerous studies indicated that the primary source of IL-33 production was astrocytes and microglia, rather than neurons (Hayakawa et al., 2007; Suzukawa et al., 2008; Wicher et al., 2017). Other studies revealed that besides in astrocytes and microglia, IL-33 was also expressed in neurons and oligodendrocytes (Pushparaj et al., 2009; Yasuoka et al., 2011). In addition to normal cellular sources, IL-33 could also be released after cellular injury or necrosis (Moussion et al., 2008), and its synthesis, localization, and release might be associated with its dual functional role. Intriguingly, IL-33 is a double-edged sword and plays a crucial role in such diseases as asthma, rheumatoid arthritis, atherosclerosis (Miller et al., 2008; Gao et al., 2017), which acts either as an anti-inflammation mechanism (Liew, 2012), or as a pro-inflammation pathway (Alves-Filho et al., 2010). Although the activity of IL-33 in diverse diseases has been well characterized, relatively little is known about the role and underlying mechanism in recurrent neonatal seizure (RNS). In this study, we established a rat RNS model and sought to determine the role of IL-33.

## Materials and Methods

### Animal Model, Reagents and Experimental Groups

All experimental procedures were in compliance with the NIH Guide for the Care and Use of Laboratory Animals and approved by the Institutional Animal Care and Use Committee at Soochow University and Wenzhou Medical University. Attempts were made to reduce animal suffering and the number of animals used. The procedure of RNS induction had been described in detail previously (Ni et al., 2005). Briefly, on postnatal day 7 (P7), 36 Sprague-Dawley (SD) rats were assigned randomly to three groups: Sham group, RNS plus PBS and RNS plus IL-33 group. From P7, rats in the RNS group were subjected to recurrent seizures induced by inhaling volatile flurothyl (Aldrich-Sigma, Chemical, USA) two times each day for consecutive 7 days, with an interval time of 30 min once. While rats in the Sham group were placed into the container for an equal amount of time to their counterpart without exposuring to flurothyl or IL-33. RNS group rats were received intraperitoneal injections of recombinant mouse IL-33 (rmIL-33, 300 ng/rat; Peprotech) or PBS 30 min before, every other day for three times, respectively. At 24 h after the last flurothyl treatment, half of the rats were anesthetized with chloral hydrate and blood samples were obtained from direct puncture of the heart with a heparinized syringe. Then the brain was removed and stored at −80°C for further analyses.

### Double Immunofluorescent Staining

Immunofluorescent analysis was performed to identify IL-33 and ST2L IR cell type in brain by standard methods, as described previously (Gao et al., 2017). Briefly, brains were perfused pericardially with PBS followed by 4% paraformaldehyde and cut into 10-μm sections with a cryotome. The sections were incubated with primary antibody with anti-IL-33 (1:100; R&D), anti-ST2L (1:200; Abcam), anti-NeuN (1:200; Abcam), anti-GFAP (1:500; Abcam), anti-Iba-1 (1:50; Abcam), then followed by a mixture of fluorescein isothiocyanate and tetra methyl rhodamine isothiocyanate conjugated-conjugated secondary antibodies for 2 h at 4°C, respectively.

### Neurobehavioral Tests

Neurological behavioral parameters of brain damage (forelimb suspension test, negative geotactic test and cliff avoidance test) were observed on P23 and P28, according to the procedure described previously (Ziegler et al., 2002; Rothstein et al., 2008). (1) Forelimb suspension test: rats were permitted to grasp a thin glass rod with their forepaws at P23 and P28, and the time required for them to remain suspended using only their forepaws was recorded. (2) Negative geotactic test: rats were permitted to place with their head down on a 45° angle slope, and the time required for them to turn 180° and to face up the slop was recorded. (3) Cliff avoidance test: rats were permitted to place on the edge of a 1.8 m high table with their forelimbs, and the time required for them to turn around and crawl away from the edge was recorded.

### Morris Water Maze Test

During the 5 days (P31–P35), the Morris water maze (MWM) test was performed to assess spatial learning and memory ability after RNS as described previously (Ni et al., 2012; Mychasiuk et al., 2015). In brief, for the place navigation test, each rat was provided with 60 s to find and climb onto the submersed platform. Once the rat found the submersed platform, it would be permitted to remain on the platform for an additional 10 s. Those rats that failed to find the submersed platform in the given time frame would be gently guided to the platform for 10 s to identify spatial cues that could be utilized in the next trial. The rats were dried by using a heat lamp before being returned to the cage. The escape latency was automatically recorded by a video/compute system. For the spatial probe test, the submersed platform was removed from the pool on P37. Then each rat was allowed to explore the pool within 60 s and the frequency of passing through the target quadrant was recorded by a video tracking system.

### Cytokine Enzyme-Linked Immune-Sorbent Assay (ELISA)

The quantitative analysis of cytokines was performed in the supernatants of brain homogenates and serum by using TNF-α or IL-1β ELISA kits (SHANGHAI BOYUN BIOTECH CO. LTD., Shanghai, China), according to the manufacturers’ protocols. In brief, reagents, samples and standard dilutions were prepared. Added 100 μL of Standard, Control, or sample per assigned well. Mixed by gently tapping the plate frame for 1 min and incubated for 1 h at 37°C. Aspirated each well and washed, repeating the process four times for a total of five washes. Then added 100 μL of Mouse TNF-α or IL-1β Conjugate to each well and incubated for 1 h at 37°C. After washing, Added 100 μL of Substrate Solution to each well with 20 min of incubation in the dark, the reaction was stopped by adding 100 μL of stop solution. Absorbance was read at 450 nm. Determinations were performed in triple, and results were expressed as mean OD ± SEM.

### Western Blot Analysis

Western blot analysis was performed to assess protein levels of apoptosis and autophagy-related proteins in the brains of RNS and control, using our standard methods as described previously (Gao et al., 2017). In brief, proteins were extracted from brain tissue and equal amounts of protein were separated by gel electrophoresis and transferred onto Hybond-polyvinylidenedifluoride (PVDF) membranes. After incubating with primary antibodies to anti-IL-33 (1:500, R&D), anti-NF-κB (1:500, CST), anti-ST2L (1:500, abcam), anti-LC3B (1:3000, abcam), anti-Beclin-1 (1:1000, bioworld), anti-P62 (1:1000, abcam), anti-cleaved-caspase-3 (1:500, bioworld), anti-Bcl-2 (1:500, abcam) and anti-β-actin (1:10,000, Sigma). Anti-β-actin was used as a loading control. Then, the PVDFs were incubated with the respective HRP-conjugated secondary antibody for 2 h at room temperature. Blots were detected with the ECL chemiluminescence system (Beyotime Institute of Biotechnology) and were captured on autoradio graphic films (Kodak). Films were scanned and densitometric analysis of the bands was performed with Sigma Scan Pro 5.

### Statistics Analysis

All the experiments were randomized and performed in a blinded manner. The data of body weight gain (BWG) and escape latency of spatial probe test in the water maze were analyzed using two-way analysis of variance (ANOVA; for subject factor and time) for repeated measures. The behavioral data and the frequency of passing through the platform quadrant of spatial probe test were carried out by one-way ANOVA with a Bonferroni test. The data of ELISA and western blot were analyzed using one-way ANOVA analysis followed by *post hoc* Tukey’s test and Dunnett *t*-test for multiple comparisons, respectively. Results were presented as means ± standard error of the mean (SEM). For all comparisons, *P* < 0.05 was regarded as statistical significant significant. All statistical analyses were performed using SPSS statistical package (version 13.0 for Windows, SPSS Inc., USA).

## Results

### The Changes of IL-33 and ST2L Expression after RNS

**Figure 1:**
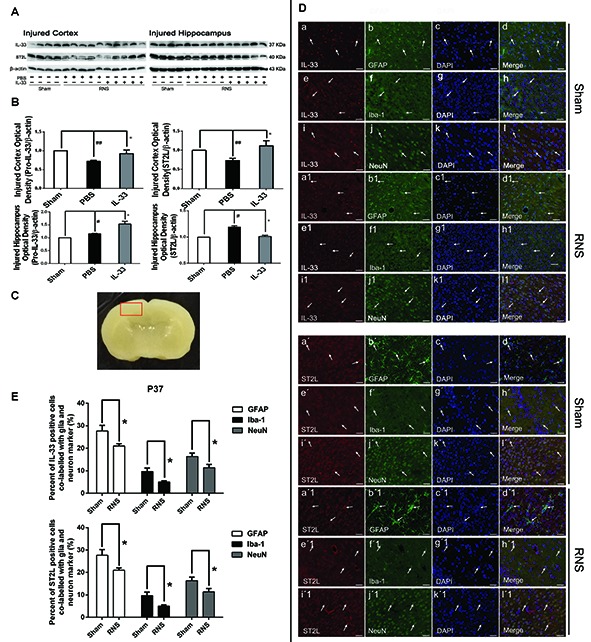
The changes and co-localization of IL-33 and ST2L expression after recurrent neonatal seizure (RNS). **(A)** The changes of IL-33 and ST2L expression in brain after RNS. **(B)** Optical densities of the protein bands were quantitatively analyzed. **(C)** Sample of double immunofluorescence staining was detected in the brain cortex tissue outlined by the red line. **(D)** Examples of double labeling are indicated with white arrows. **(d,d_**1**_)** Co-localization of IL-33-like immnoreactivity and GFAP. Bar 50 μm. **(h,h_1_)** Co-localization of IL-33-like immnoreactivity and Iba-1. Bar 50 μm. **(l,l_1_)** NeuN-positive neurons were positive for IL-33. Scale bar 50 μm. **(d′,d′_1_)** Co-localization of ST2L-like immnoreactivity and GFAP. Bar 50 μm. **(h′,h′_1_)** Co-localization of ST2L-like immnoreactivity and Iba-1. Bar 50 μm. **(l′,l′_1_)**. The co-localization of strong ST2L-like immnoreactivity and NeuN. Bar 50 μm. **(E)** Semi-quantitative analysis of glia or neuron type-cell contributions to the IL-33 or ST2L positive cell population. The data were expressed as means ± SEM (*n* = 4–6). ^##^*P* < 0.01 vs. Sham group, ^#^*P* < 0.05 vs. Sham group. *P < 0.05 vs. PBS group. Experiments are representative of three independent experiments.

To investigate the effect of IL-33 on RNS, we detected the level of IL-33 and its receptor ST2L in brain cortex and hippocampus (Figures 1A,B). We found that RNS contributed to significant reduction in IL-33 and ST2L expression in cortex. While, in hippocampus, RNS induced an increase in IL-33 and ST2L evidently, compared with Sham group. However, after injection with IL-33, a remarkable increase in total IL-33 was detected both in brain cortex and hippocampus, implying that rmIL-33 has been arrived at the site of the injured brain parenchyma. Furthermore, a striking increase in ST2L in cortex and an evident reduction in ST2L in hippocampus were induced by rmIL-33 pretreatment.

### Identification of IL-33 Immuno-Reactive Cell Type in Brain

To define the phenotype of the IL-33 immune-reactive (IR) cells after RNS, we analyzed co-localization of IL-33 with specific markers for astrocytes (GFAP), microglial (Iba-1) or neurons (NeuN) in cerebral cortex (Figure 1C), respectively. Our results indicated that IL-33 was co-expressed in GFAP^+^ astrocytes, and mainly displayed nuclear staining (Figures 1Dd,d_1_). Novelty, IL-33 expressed exclusively in cytoplasm of the Iba-1^+^ microglia, rather than in nucleus (Figures 1Dh,h_1_). In parallel, IL-33 was also present in the cytoplasm of IL-33^+^/NeuN^+^ merged cells both in Sham group and RNS group (Figures 1Dl,l_1_). Furthermore, quantitative analysis of the above-mentioned three types of double-labeled cells was obviously reduced in RNS group, compared with Sham group (Figure 1E). Thus, these results indicated that IL-33 was expressed in astrocytes and microglia, as well as in neurons after RNS.

### Identification of ST2L Immuno-Reactive Cell Type in Brain

In order to identify the phenotype of the ST2L IR cells in model of RNS, we investigated co-expression of ST2L with specific markers for astrocytes (GFAP), microglial (Iba-1) or neurons (NeuN) in cerebral cortex (Figure 1C), respectively. We detected co-localization of ST2L with GFAP^+^ astrocytes (Figures 1Dd′,d′_1_), Iba-1^+^ microglia (Figures 1Dh′,h′_1_) and NeuN^+^ neurons (Figures 1Dl′,l′_1_) both in Sham group and RNS group, respectively. Moreover, RNS group significantly induced quantitative analysis of the above-mentioned three types of double-labeled cells, compared with Sham group (Figure 1E).

### IL-33 Improves Neurologic Behavioral Deficits and Promotes Body Weight Gain after RNS

**Figure 2:**
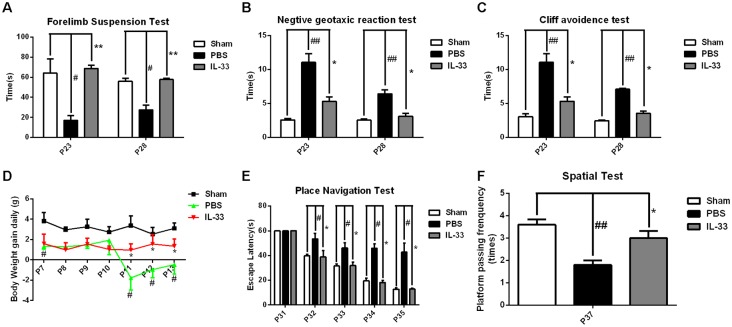
IL-33 improves neurologic behavioral deficits and promotes body weight gain (BWG) after RNS. **(A–C)** Times of each group in forelimb suspension test, negative geotactic test and cliff avoidance test at P23 and P28, respectively. **(D)** IL-33 reversed RNS-induced the reduction in BWG. **(E)** In morris water maze (MWM) test, mean escape latency for each group was plotted during P31–P35. **(F)** The frequencies of crossing the platform were recorded at P37. The data were expressed as means ± SEM (*n* = 4–6). ^##^*P* < 0.01 vs. Sham group, ^#^*P* < 0.05 vs. Sham group. **P < 0.01 vs. PBS group, *P < 0.05 vs. PBS group. Experiments are representative of three independent experiments.

To assess the effect of IL-33 on neurologic development and BWG, different neurological tests were presented (Figures 2A–C). We found that RNS group had an evident delay or reduction of forelimb suspension test, negative geotactic reaction test and cliff avoidance test, compared with Sham group. In contrast, these behavioral deficits were reversed by IL-33 pretreatment, compared with RNS group. Besides, we examined the temporal changes of BWG from P7 to P13. A significant reduction in BWG was observed at P7 post-RNS. The reduction reached the valley at P11, and increasingly returned to baseline levels, but remained the decreased levels for up to P13. However, IL-33 pretreatment obviously promoted BWG from P11 to P13, suggesting that IL-33 may exert neuroprotection via promoting BWG following RNS (Figure 2D).

### IL-33 Ameliorated Performance in Morris Water Maze (MWM) Test after RNS

As shown in Figures 2E,F, the escape latencies of MWM were significantly longer in RNS group from P31 to P35 than that in Sham group; however, the latency was significantly reduced in IL-33 pretreatment group, compared with RNS group. As far as spatial probe test was concerned, the frequency of passing through the platform quadrant was significantly lower in RNS group than that in Sham group. Whereas, this significant decrease in the probe tests was reversed by IL-33 pretreatment, compared with RNS group.

### The Anti-Inflammatory Cytokine Role of IL-33 in RNS

**Figure 3:**
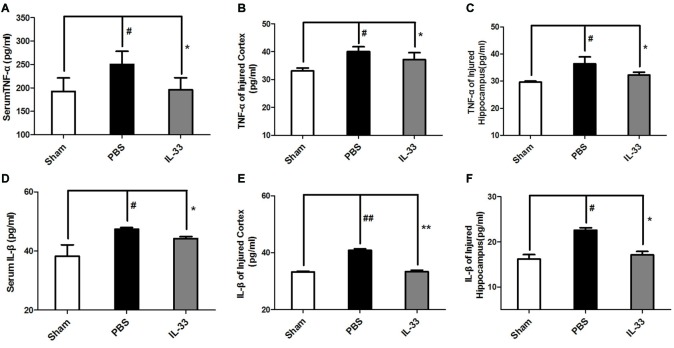
IL-33 is an anti-inflammation cytokine after RNS. The expression levels of IL-1β and TNF-α were measured by enzyme-linked immune-sorbent assay (ELISA). RNS-induced TNF-α and IL-1β increase were reversed by IL-33 pretreatment among serum **(A,D)**, brain cortex **(B,E)** and hippocampus tissues **(C,F)** after seizure. The data were expressed as means ± SEM (*n* = 4–6). ^ ##^*P* < 0.01 vs. Sham group, ^#^*P* < 0.05 vs. Sham group. **P < 0.01 vs. PBS group, *P < 0.05 vs. PBS group. Experiments are representative of three independent experiments.

To demonstrate whether IL-33 affects inflammatory responses after RNS. ELISA was conducted to assess the level of inflammatory cytokines. The results indicated that IL-1β and TNF-α expression were significantly increased in RNS group, compared with Sham group. On the contrary, up-regulation of IL-1β and TNF-α in serum (Figures 3A,D), brain cortex (Figures 3B,E) and hippocampal tissues homogenates (Figures 3C,F) were reversed by IL-33 pretreatment, compared with the RNS group.

### IL-33 Reversed RNS-Induced NF-κB Activity

**Figure 4:**
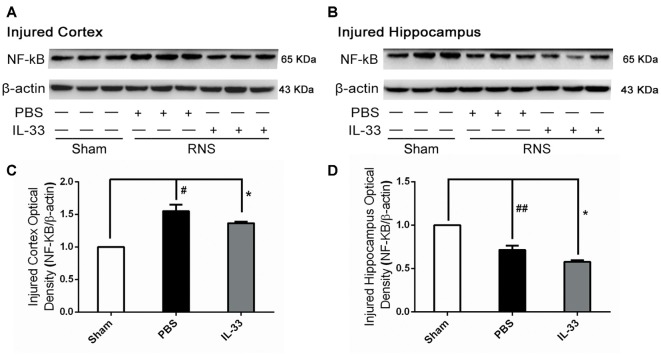
IL-33 reversed the RNS-induced NF-κB activity increase. **(A,B)** After RNS, a significant increase of NF-κB activity was detected both in brain cortex and hippocampus tissues, compared with Sham group. However, IL-33 remarkably suppressed NF-κB activity, compared with PBS group, suggesting that IL-33 possess a capacity to decline NF-κB activity following RNS. **(C,D)** Optical densities of the protein bands were quantitatively analyzed, and normalized with loading control β-actin. The data were expressed as means ± SEM (*n* = 4–6). ^##^*P* < 0.01 vs. Sham group, ^#^*P* < 0.05 vs. Sham group. *P < 0.05 vs. PBS group. Experiments are representative of three independent experiments.

To investigate the effect of IL-33 on NF-κB activation after RNS, Western blot was performed to assess NF-κB expression. Here, we observed that there were a significant increase of NF-κB activity in brain cortex, and a significant reduction in hippocampus, compared with Sham group. However, IL-33 pretreatment strikingly suppressed NF-κB activity, compared with RNS group (Figure 4), suggesting that IL-33 may possess a capacity to down-regulate NF-κB activity following RNS.

### IL-33 Increased Bcl-2 but Decreased Cleaved-Caspase (CC-3) Expression after RNS

**Figure 5:**
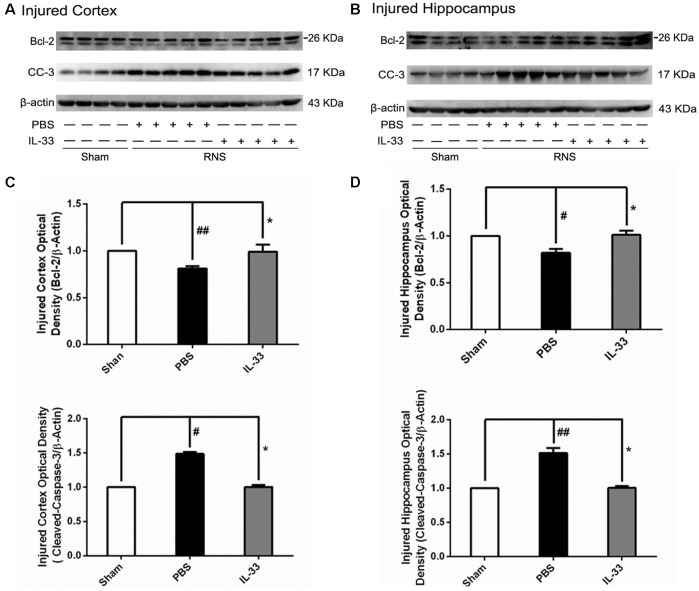
IL-33 reversed the RNS-induced Bcl-2 decrease and CC-3 increase. **(A,B)** IL-33 pretreatment inhibited RNS-induced up-regulation of CC-3 expression and down-regulation of Bcl-2 expression both in cortex and hippocampus tissues after RNS. **(C,D)** Optical densities of the protein bands were quantitatively analyzed, and normalized with loading control β-actin. The data were expressed as means ± SEM (*n* = 4–6). ^##^*P* < 0.01 vs. Sham group, ^#^*P* < 0.05 vs. Sham group. *P < 0.05 vs. PBS group. Experiments are representative of three independent experiments.

To determine the effect of IL-33 on apoptotic pathway after RNS, Western blotting was utilized to evaluate the expression levels of Bcl-2 and CC-3. As shown in Figure 5, RNS significantly induced the decrease of Bcl-2 expression and promoted CC-3 expression, compared with Sham group. Whereas, IL-33 pretreatment obviously inhibited down-regulation of Bcl-2 expression, and up-regulation of CC-3 expression both in cortex and hippocampus.

### IL-33 Reversed RNS-Induced Autophagic Activation

**Figure 6:**
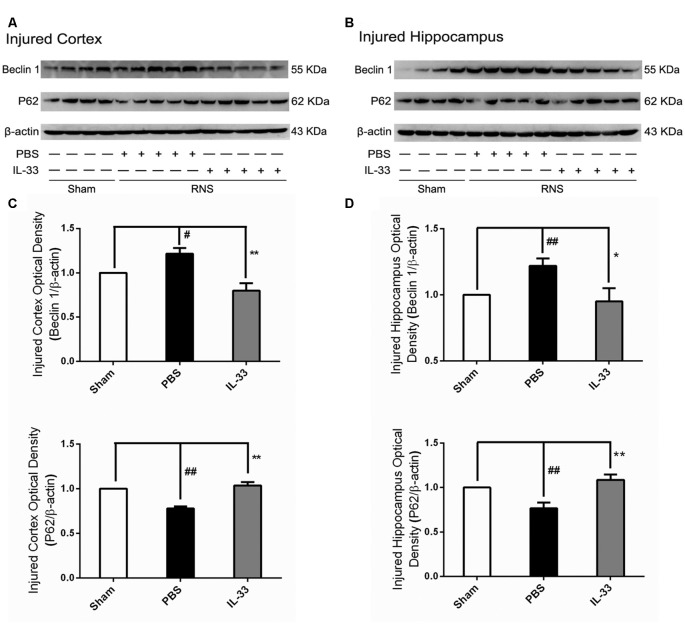
IL-33 reversed the RNS-induced increase of Beclin-1 and the decrease of P62. **(A,B)** IL-33 pretreatment significantly suppressed Beclin-1 expression and maintained p62 at a high level after RNS. **(C,D)** Optical densities of the protein bands were quantitatively analyzed, and normalized with loading control β-actin. The data were expressed as means ± SEM (*n* = 4–6). ^##^*P* < 0.01 vs. Sham group, ^#^*P* < 0.05 vs. Sham group. **P < 0.01 vs. PBS group, *P < 0.05 vs. PBS group. Experiments are representative of three independent experiments.

**Figure 7:**
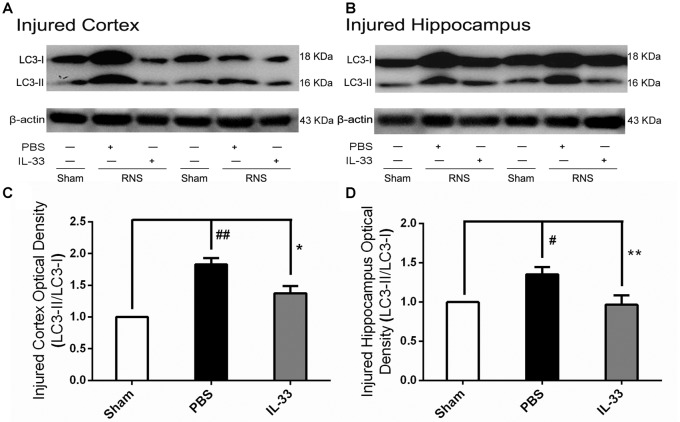
IL-33 negatively regulates autophagy after RNS. **(A,B)** IL-33 pretreatment significantly reduced the LC3-II/LC3-I ratio. **(C,D)** Optical densities of the protein bands were quantitatively analyzed, and normalized with loading control β-actin. The data were expressed as means ± SEM (*n* = 4–6). ^##^*P* < 0.01 vs. Sham group, ^#^*P* < 0.05 vs. Sham group. **P < 0.01 vs. PBS group, *P < 0.05 vs. PBS group. Experiments are representative of three independent experiments.

**Figure 8:**
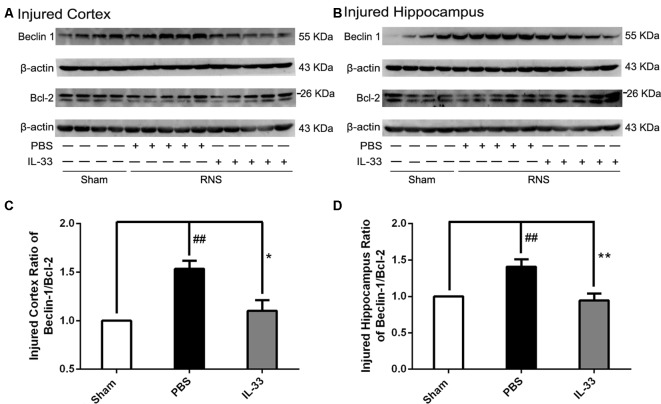
IL-33 pretreatment reversed the RNS-induced increase of the Beclin-1/Bcl-2 ratio. **(A,B)** IL-33 pretreatment down-regulated the Beclin-1/Bcl-2 ratio after RNS. **(C,D)** Optical densities of the protein bands were quantitatively analyzed, and normalized with loading control β-actin. The data were expressed as means ± SEM (*n* = 4–6). The data were expressed as means ± SEM (*n* = 4–6). ^##^*P* < 0.01 vs. Sham group. **P < 0.01 vs. PBS group, *P < 0.05 vs. PBS group. Experiments are representative of three independent experiments.

To confirm whether IL-33 plays a crucial role in autophagic activation after RNS, western blot analysis was used to assess the expression of autophagy-associated proteins such as Beclin-1, p62 (Figure 6) and LC3-II (Figure 7). Higher amounts of LC3-II/LC3-I and Beclin-1, and lower amounts of P62 were found in RNS group, compared with Sham group. However, administration of IL-33 decreased the ratio of LC3-II/LC3-I, Beclin-1 expression and maintained P62 at normal level after RNS both in brain cortex and hippocampus. Moreover, IL-33 pretreatment significantly reduced the Beclin-1/Bcl-2 ratio after RNS (Figure 8).

## Discussion

IL-33 is a chromatin-associated cytokine that has recently been linked to many diseases (Hudson et al., 2008; Miller et al., 2008; Gao et al., 2017). However, whether IL-33 plays a primary role in RNS has never been clarified yet. The study aimed at clarifying the role of IL-33 and underlying mechanism in RNS. Our results showed that IL-33 pretreatment improved RNS-induced behavioral deficits, promoted bodyweight gain, ameliorated performance in MWM test. To further investigate the molecular mechanism, we found IL-33 could block the activation of NF-κB, and thereby resist IL-1β and TNF-α increase, as well as suppress apoptosis and autophagy after RNS.

Previous study reported that IL-33 was constitutively expressed in the skin, pancreas and brain (Zakeri et al., 1995; Prefontaine et al., 2010). Moreover, IL-33 was expressed at high levels in brain (Han et al., 2011). Although the expression and localization of IL-33 and ST2L are increasingly clear, the expression changes and the role in malignant state are little known yet. Therefore, we used a rat model of RNS to demonstrate the expression changes and the functional role of IL-33 and ST2L, and subsequently to explore the potential mechanisms.

Our results indicated that RNS contributed to a significant reduction in IL-33 and ST2L expression in cortex, and an evident increase in IL-33 and ST2L in hippocampus, compared with Sham group. However, IL-33 pretreatment resulted in a striking increase in ST2L in cortex and an evident reduction in ST2L in hippocampus. Seizure can induce sever pathologies including cell death, neuronal loss and inflammatory responses, which can contribute to switching pro-IL-33 to mature IL-33 (Cayrol and Girard, 2009). Moreover, processing of full length IL-33_1–270_ by caspases results in its inactivation (Cayrol and Girard, 2009). The significant reduction of pro-IL-33 in cortex by seizure might be a result of caspase-1, caspase-3 and calpain elevation, which led to cleave pro-IL-33 into mature IL-33 and thereby resulted in low detection of pro-IL-33. Whereas, the ST2L up-regulation in cortex by IL-33 pretreatment may be due to the feedback responds of pro-IL-33 decreasing and vice versa in hippocampus. Despite of the explanations of these changes, our findings may provide an important clue to study the role of IL-33 after RNS and the underlying mechanisms.

Although the expressions and changes of IL-33 and ST2L are increasingly clear-cut, the co-localization of them with neural cell population needs to be further elucidated. Numerous literatures identified that IL-33 is produced by endothelial cells and astrocytes but not by microglia or neurons (Manetti et al., 2010; Yasuoka et al., 2011), and its receptors are mainly expressed in microglia and astrocytes *in vitro* cellar culture (Yasuoka et al., 2011). Other reports revealed that IL-33 was mainly localized in astrocytes, microglia and neurons after brain injury *in vivo* (Huang et al., 2015; Gao et al., 2017). Consistent with the latter, our current results showed that IL-33 almost exclusively located in the nucleus of astrocytes, and in the cytoplasm of microglia and neurons. In the meanwhile, ST2L, the receptor of IL-33 is co-expressed in the cytomembrane of the above-mentioned three types of neural cells after RNS. We speculate the reason for the discrepancy may be caused by the difference experiment settings, environments between *in vitro* and *in vivo*, or because of tissue repair process after acute brain injury. This intriguing observation warrants further investigation.

The crucial effect of inflammatory responses on diseases has been consecutively focused on. As a novel cytokine, IL-33 is a double-edged sword and exerts a critical role in numerous diseases (Liew, 2012). However, the effect of IL-33 on inflammatory responses following RNS has never been reported yet. Previous findings suggested that seizure activity led to the production of inflammatory molecules including IL-1β and TNF-α (Vezzani et al., 2011) that, in turn, affected seizure severity and recurrence (Ichiyama et al., 1998; Auvin et al., 2010). While, suppression of IL-1β or TNF-α expression could lead to prevention or delay of seizures, and exerts neuroprotection in a rat model of temporal lobe epilepsy (Noe et al., 2013; Sitges et al., 2014). Intriguingly, in the study, IL-33 pretreatment significantly suppressed inflammatory responses through reducing IL-1β and TNF-α expression both in serum and brain tissues homogenates. Moreover, IL-33 pretreatment also improved behavioral deficits, promoted bodyweight gain, ameliorated performance in MWM test after RNS. Based on these findings, we can envisage that IL-33 may exert neuroprotective role in RNS through anti-inflammatory pathway. However, very little is known about the underlying mechanisms of IL-33 in dampening inflammatory cytokines IL-1β and TNF-α expression after RNS.

Previous study demonstrated that NF-κB plays a critical role in chronic inflammatory diseases and its activation is essential for cytokine production (Luedde and Schwabe, 2011). On the one hand, NF-κB activation can regulate transcription and expression of genes encoding cytokines. On the other hand, the blockage of NF-κB activation can inhibit the release of pro-inflammatory cytokine (Luedde and Schwabe, 2011). Consistently, our results indicated that IL-33 pretreatment led to an evidently down-regulation of NF-κB expression, and accompanied by down-regulation of pro-inflammatory cytokines levels after RNS, implying that IL-33 may possess a capacity to inhibit pro-inflammatory signaling at least in part by blocking NF-κB activation. Further studies are required to evaluate this in more detail.

Seizure could result in multiple types of cell death such as apoptotic, autophagic cell death and necroptosis (Lopez-Meraz et al., 2010; Benz et al., 2014). Moreover, seizure-induced neural cell apoptosis is associated with proteolytic activation of effector caspases (Engel et al., 2010). Among these identified apoptotic genes, Bcl-2 and caspase-3 are widely recognized as the most important apoptotic regulators, and their relative levels determine the fate of neural cells. Previous study demonstrated that IL-33 not only significantly reduced hepatocyte apoptosis by enhancing Bcl-2 expression (Sakai et al., 2012), but also attenuated MC apoptosis through enhancing Bcl-xl (Wang et al., 2014). Consistent with previous studies, our recent results indicated that IL-33 pretreatment significantly enhanced Bcl-2 and inhibited caspase-3 expression, suggesting an effective role in rescuing neural cell apoptosis. Collectively, IL-33 blocks cell apoptosis via increasing Bcl-2 and reducing caspase-3 expression after RNS.

In addition to inhibiting apoptosis, the role of IL-33 in RNS-induced autophagy activation remains to be determined. Our recent results indicated that IL-33 pretreatment reversed RNS-induced autophagic activation by reducing the LC3-II/LC3-I ratio, Beclin-1 expression and maintaining P62 at high level. Previous studies revealed that autophagy represents a double-edged sword, acting either as a pro-survival mechanism, or as one of cell death pathways (Apel et al., 2009; Martinet et al., 2009). That is, the effect of autophagy may be dictated by whether or not it can meet intracellular demands. Moreover, our previous studies demonstrated that inhibition of autophagy provided neuroprotection in multiple experimental models of brain injury (Zhang et al., 2014; Gao et al., 2017). The above findings revealed that inhibition of autophagy might respect a novel and promising tool in the pretreatment of diseases of the nervous system. Overall, IL-33 dampened autophagy, which might be associated with the regulation of autophagic related proteins after RNS. No matter, further studies are needed to determine the underlying mechanisms of IL-33 regulating autophagy after RNS. The dynamics of complex biological processes between apoptosis and autophagy may be one of the reason to explain the regulatory effect of IL-33 on autophagy after RNS. On the one hand, the antiapoptotic protein, Bcl-2 can suppress apoptosis by directly binding the pro-apoptotic effector proteins Bax, Bak and Bim via the BH3 binding groove (Lindqvist et al., 2014). On the other hand, it can function as a brake on autophagy via binding to Beclin-1’s BH3 domain (Pattingre et al., 2005; Satoo et al., 2009). Therefore, the anti-autophagic function of IL-33 may be partially illustrated that the abundance of Bcl-2 sequestered free Beclin-1, and then down-regulated the autophagic activation via disrupting the formation and activity of the autophagy-promoting Beclin-1/hVps34 complex. Furthermore, promoting autophagy also regulates cellular loss against apoptosis through up-regulation of the Bcl-2/Bax ratio (Luo et al., 2011; Jiang et al., 2014), and the ubiquitin-binding protein p62/SQSTM1 (p62) promotes cullin3-modified caspase-8 aggregation within p62-dependent foci, which can lead to full activation of the enzyme and then to control apoptosis signaling positively (Jin et al., 2009). Therefore, it might be speculated that IL-33 inhibited apoptosis and thereby blocked autophagic activation or vice versa, or directly inhibited both apoptosis and autophagy by promoting Bcl-2 expression after RNS.

Taken together, the current data demonstrates that IL-33 provides neuroprotection against RNS-induced brain injury through suppressing apoptosis, autophagy and at least in part by NF-κB-mediated inflammatory pathways, and IL-33 may be a potential therapeutic agent for RNS.

## Author Contributions

YG, CL and LL performed the experiments and analyzed data. YG, HN and LT is responsible for the conception and design of the study and manuscript writing. YG, CL, GY, CG, TW and ZW performed the animal experiments. HW and WH helped to process data. YG, XC and LT contributed to manuscript preparation.

## Conflict of Interest Statement

The authors declare that the research was conducted in the absence of any commercial or financial relationships that could be construed as a potential conflict of interest.
